# A Novel Health Indicator Based on Cointegration for Rolling Bearings’ Run-To-Failure Process

**DOI:** 10.3390/s19092151

**Published:** 2019-05-09

**Authors:** Hongru Li, Yaolong Li, He Yu

**Affiliations:** Army Engineering University, No. 97 Heping West Road, Shijiazhuang 050003, China; bywddt@163.com (Y.L.); 13832329446@163.com (H.Y.)

**Keywords:** rolling bearings, degradation model, generalization, vibration signal

## Abstract

The extraction of rolling bearings’ degradation features has been developed for decades. However, the degradation features always present different trends of different run-to-failure data. To find a consistent indicator of different data will be helpful to establish a general model and explore the nature of bearings’ degradation. In this study, we have found there is a trend of similarity between the energy and complexity features. By using the cointegration test, we found the two kinds of features exhibit a certain degree of cointegration relationship. Fused by the cointegration method, we have obtained a novel health indicator which can depict different run-to-failure data in a unified way. The difference between the energy features and complexity features can be explained by the novel health indicator. The indicator has “two-stage” characters. The first stage is the zero-line stage and the second stage is the quickly raise stage, which presents like an exponential function. It is easy to think about using an exponential degradation model to model this indicator. Next, we have compared the indicator to root mean square (RMS) by using the exponential degradation model. It shows that the indicator is more suitable for the exponential degradation model. In this paper, we used eleven run-to-failure data to verify the generality and “two-stage” characters of the proposed indicator. The result shows that the novel indicator is general and effective and that it will promote the development of bearings’ prognostics.

## 1. Introduction

The rolling bearing is one of the most important components in rotating machinery. As one of the most widely used and easily vulnerable parts in rotating machinery, its running state is directly related to the overall performance of the equipment. So, clearly understand the running state of bearings and accurate prediction of rolling bearings’ remaining useful life is very important in industry. Thus, the concept of condition-based maintenance (CBM) was proposed to monitor the degradation process and predict the remaining useful life of bearings. In practice, the most extensively used approach for CBM is vibration signals.

Nowadays, many researchers are devoted to find, and have already proposed many degradation features which give a good representation of bearings’ degradation process. There are some excellent review articles which summarize and classify the proposed degradation features. In [1], Lei et al. categorized the degradation features into two classes according to their construction strategies: physic ones and virtual ones. Physic ones are the features which are related to the physics of failures and generally extracted from monitoring signals using statistical methods or signal processing methods. Virtual ones are the features which are constructed by fusing methods. In [2], Wang et al. reviewed the bearing and gear health indicators and categorized them into three classes: mechanical signal processing-based, model-based and machine learning-based. Both in [3] and [4], the features are classified into time-domain features, frequency-domain features and time–frequency domain features. In the time-domain, the features are generally measure statistical characteristics of signals. The frequency-domain is based on a transformation of signal into frequency domain. The fast Fourier transform (FFT) is the foundation of frequency-domain analysis. The time–frequency domain features are extracted based on the different signal processing methods. In recent years, the signal processing methods for degradation features have developed rapidly, e.g., Wavelet Transform (WT) [5], Empirical Mode Decomposition (EMD) [6], Ensemble Empirical Mode Decomposition (EEMD) [7], Local Mean Decomposition (LMD) [8], Variational Mode Decomposition (VMD) [9], Empirical Wavelet Transform (EWT) [10], etc.

However, the traditional proposed degradation features are lacking of a consistent trend, and the degradation features of different run-to-failure data of bearings often do not have a unified degradation law. If the features follow a sort of consistent degradation trend of different data, it will be helpful to establish a general model and explore the nature of bearings’ degradation. Therefore, we are going to find a novel health indicator which could build a general degradation model for rolling bearings’ prognostics.

Among the aforementioned reviews, there are two degradation features which have been referred to, one is the energy features, the other is the complexity features, where the root mean square (RMS) and the Sample Entropy (SampEn) are two representatives. To show the fact that different run-to-failure data of bearings do not have a unified degradation trend, an example is presented below.

**Figure 1:**
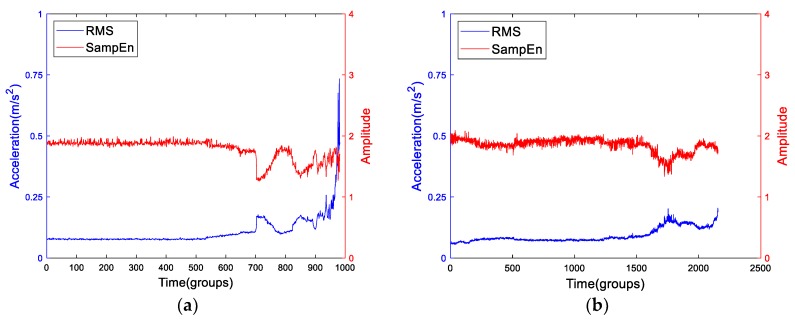
The root mean square (RMS) and SampEn curves of (**a**) Bearing2-1IMS and (**b**) Bearing1-4IMS.

The Intelligent Maintenance System (IMS) Center of the United States has carried out the bearing life test and provided the original vibration signal dataset [5], which are widely used in many references. In this paper, we called this dataset ‘Dataset I’. The details of the dataset we used in the paper are presented in the Appendix A. In Dataset I, the first data of the second test which is named Bearing2-1IMS were the frequently-used public data in many papers [11,12,13,14]. The failure mode of Bearing2-1IMS was outer race fault and the data contains 984 group samples. When we carefully checked the last two group samples, we found both of them were periodic signals. Actually, they did not belong to the run-to-failure data. So, the Bearing2-1IMS data included 982 effective groups. Taking this data as an example, we can plot the RMS and SampEn curves as shown in Figure 1a. Meanwhile, we have plotted the RMS and SampEn of Bearing1-4IMS in which the failure mode was roller element fault as a comparison.

From Figure 1, we can see that for different run-to-failure data of bearings, the RMS and SampEn vary greatly. At the same time, the features are not completely monotonous, i.e., there exists many up and downs, this is a so-called ”healing” or smoothing phenomenon [15,16], and it is not good for prognostics. We can see that for an individual bearing, the RMS and SampEn have a certain opposite trend, especially in the long period before the bearings are close to failure. In economics, there is a term named cointegration, we can conjecture that there exists a possible cointegration relationship between the RMS and SampEn. In this paper, we are going to fuse the two degradation features by introducing the cointegration theory and propose a general health indicator which exhibits a character of “two-stage” property. Thus, volatility of traditional degradation features will be reduced and the ability of fault prediction will be increased. In this paper, many run-to-failure data have been applied to verify the generalization of the proposed model.

The remainder of this paper is composed as follows: In Section 2, the concept of cointegration and the method to test cointegration has been introduced. In Section 3, we will investigate the energy and complexity features in detail. By comparisons, we can test their performance and find a better representative of each subdivision. In Section 4, we are going to propose the novel health indicator by fusing the two features with cointegration theory and we will explain the reason of the “two-stage” stages. With many run-to-failure data, we can verify the proposed model. In the discussion section, we are going to talk about the significance of the cointegration fused method and the difference between other fused methods. Finally, concluding remarks are given in Section 6.

## 2. The Methodology of Cointegration

### 2.1. The Stationary Process and Unit Root Test

Before talking about cointegration some terms should be clarified. In statistics, there are two kinds of stationary process. One is the strict stationary process, the other is weak stationary process [17]. In general, the stationarity of a time-series refers to the weak stationary process.

The unit root test can test whether a time-series is stationary or not. When the unit root exists, it means the time-series is non-stationary. A commonly used unit root test is augmented Dicky–Fuller (ADF) test. The ADF test is an extension of Dicky–Fuller test which introduced lags of the order p so that it can apply to higher-order autoregressive processes. For a *p*-order autoregressive model AR(*p*),

(1) $$y_{t}=c+\alpha_{1}y_{t-1}+\alpha_{2}y_{t-2}+\cdots +\alpha_{p}y_{t-p}+\varepsilon_{t}\Leftrightarrow ∆y_{t}=c+\rho y_{t-1}+\sum_{i=1}^{p-1}\phi_{i}∆y_{t-i}+\varepsilon_{t}$$

where c is a constant, p is the lag order of autoregressive model, $\rho =(\sum_{i=1}^{p}\alpha_{i})-1$ is the coefficient of the time trend, $\phi_{i}=-\sum_{j=i+1}^{p}\alpha_{j}$, and $\varepsilon_{t}$ is the white noise. The unit root test is carried out under the null hypothesis ρ=0 against the alternative hypothesis of ρ<0. Once a value for the test statistic is computed, it can be compared with the relevant critical value for the ADF test. If the test statistic is less than the critical value, then the null hypothesis is rejected which means there is no unit root of the time-series.

There are three versions of the test, which are displayed below.

1st version: $∆y_{t}=\rho y_{t-1}+\sum_{i=1}^{p-1}\phi_{i}∆y_{t-i}+\varepsilon_{t}$

2nd version: $∆y_{t}=c+\rho y_{t-1}+\sum_{i=1}^{p-1}\phi_{i}∆y_{t-i}+\varepsilon_{t}$

3rd version: $∆y_{t}=c+\gamma t+\rho y_{t-1}+\sum_{i=1}^{p-1}\phi_{i}∆y_{t-i}+\varepsilon_{t}$

**Figure 2:**
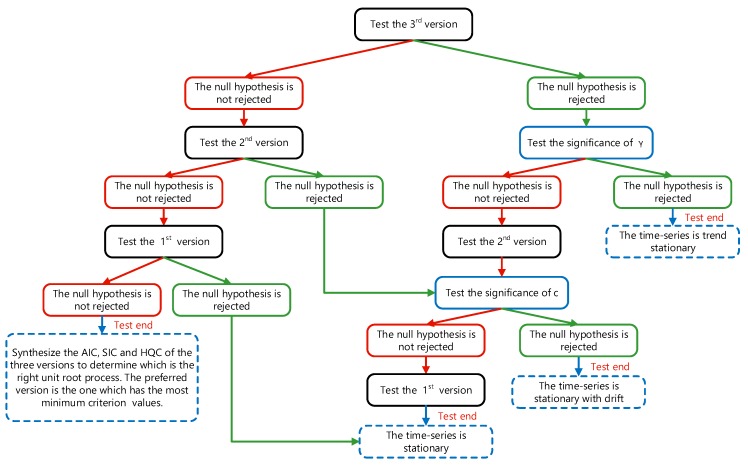
The flow chart of the augmented Dicky–Fuller (ADF) test.

The three versions are tests for a unit root, tests for a unit root with drift and tests for a unit root with drift and deterministic time trend, respectively. When conducting the ADF test, the lag order *p* should be determined. A good approach is to examine information criteria such as the Schwarz information criterion (SIC), the Akaike information criterion (AIC) and the Hannon–Quinn information criterion (HQC). The flow chart of the ADF test is depicted in Figure 2. More details of the ADF test can be seen in [18,19].

### 2.2. The Order of Integration

There is another term named the order of integration, denoted as I(d), which represents the minimum number of differences required to obtain a stationary series. For a time-series integrated of order d, it requires that ${\left(1-L\right)}^{d}X_{t}$ is stationary, where L is the lag operator and 1−L is the first difference.

### 2.3. Cointegration

The cointegration is a statistical property of a collection of time-series which was coined by Engle and Granger in 1987 [20]. The concept of cointegration describes the long-run linear equilibrium relationship among a collection of time-series. First, all the time-series must be I(d), that is a premise. The aim of cointegration is to find if their linear combination has a lower order of integration. Indeed, the cointegration yields a reduction of the order of integration. If the linear combination of these time-series had a lower order of integration valued b, then we called these series cointegrated of order b, denoted as CI(d,b). If d=b>0, we called them cointegrated. For example, if two time-series X,Y are all I(3), and there exists coefficients a,b require that aX+bY is I(2), then we called X, Y as having the relationship of CI(3,2) and (a,b) as the cointegration vector. In general, CI(1,1) is the most common cointegration relationship. If two sequences have a cointegration relationship, then they have the same trend.

There are two main methods for testing cointegration: one is the Engle–Granger method [20], the other is the Johansen test [21]. The Johansen test is generally used for multiple time-series. The Engle–Granger test is specifically used for two sequences. The process of Engle–Granger test is simply stated as below. If $x_{t}$ and $y_{t}$ have a cointegration relation with CI(1,1), then the linear combination of them is stationary, i.e.,:

(2) $$y_{t}-\alpha_{1}x_{t}=e_{t}$$

where $e_{t}$ is the residual. If we know $e_{t}$, we can just test if it is stationary by using theADF test. However, if we don’t know $e_{t}$ at the beginning, we must estimate it first, generally by using the ordinary least squares. Then we may run the stationarity test on the estimated $e_{t}$ series, namely ${\hat{e}}_{t}$ to test if there exists unit roots.

## 3. The Energy Features and Complexity Features

The energy and complexity features are two subdivisions of the degradation features of rolling bearings. The energy features have an uptrend of the run-to-failure data. When the fault deepens, the stress concentration will be more obvious, thus leading to an increase in energy features. Meanwhile, when the fault deepens, the vibration signals will be more periodic, thus leading to a decrease in complexity features. In this section, we are going to investigate the energy and complexity features in detail and compare them in each subdivision.

### 3.1. The Energy Features

First of all, a clear understanding of what a degradation feature is must be made. A degradation feature must be extracted from the run-to-failure data. We must make sure that the data are obtained from a normal bearing to a faulty bearing with the increasing of running time to make sure that the fault propagates in a natural way. If we make some damages to the normal bearing, like slitting a slot on the outer race and carry out the test, then when the slot expands to a threshold, we stop running the test. This procedure is a preset fault test and commonly applied for fault diagnosis, but it could not be deemed as a run-to-failure process.

**Figure 3:**
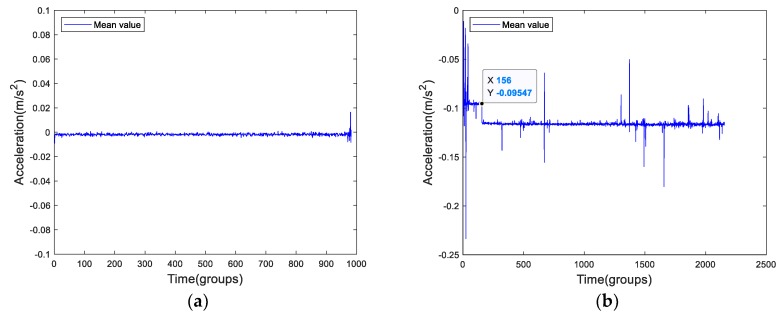
The mean value of (**a**) Bearing2-1IMS and (**b**) Bearing1-4IMS.

Generally, each group of data can extract a degradation feature point. All the points constitute a time-series as a degradation feature. It is only when the features have a trend that we can assess the degradation performance and predict the remaining useful life (RUL). Taking a counterexample, the mean value could not be considered as a degradation feature. Figure 3 shows the mean value of the two examples in the introduction.

**Figure 4:**
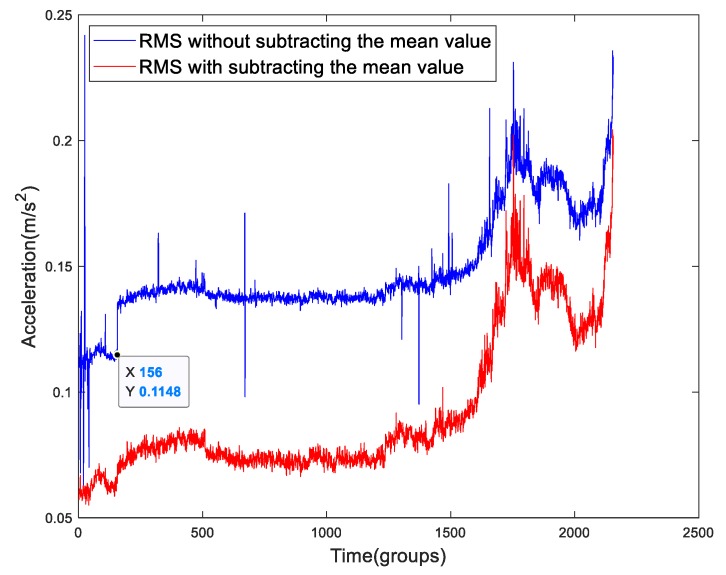
The Bearing1-4IMS’s RMSs with and without subtracting the mean value.

As we can see, in Figure 3a, the mean value is almost zero with a slight fluctuation close to failure. This is a normal phenomenon of the mean value for a run-to failure data. The mean value should be around zero. When the degradation is close to failure, the violent vibration may cause the jitter of the mean value. However, in Figure 3b, there are more fluctuations in the feature, especially a step drop at 156th group. It is an abnormal phenomenon. The values of this time-series are less than zero, which suggests that the sensor may not undertake zero alignment or the bearings and shaft were not properly installed before the test. The degradation feature extracted from the original data will be influenced by the fluctuation particularly a step change of the mean value. To give a better comprehension of the effect of the mean value, a comparison of Bearing1-4IMS’s RMSs with and without mean removal is shown in Figure 4. We can see an inshot at 156th group of the RMS without mean removal. A few of references have neglected this process. For example, in Figure 20 of [11], we can see a step raise at about 120th group that was the cause of the fluctuation of mean value. When we calculated the degradation features in this paper, we subtracted the mean value to eliminate such effects.

Back to the subject, the mean value could not be deemed as a degradation feature because we could not assess the degradation stage through its process. In our opinion, a degradation feature should have an uptrend or downtrend. For uptrend features, the largest branch should be energy features. In Table 1, some energy features are listed. The feat1 to feat6 features were the amplitude of root, the RMS, the absolute mean value, the peak-to-peak value, the maximum value and the minimum value, respectively. For convenience, there is a minus sign before the minimum value to make it positive. Feat1 to feat6 were all time-domain features. They measured the statistical indicators in the time domain.

Why are the aforementioned features all energy features? Let’s simplify the model. Take a vibration particle at a certain moment as a simple harmonic oscillation. The displacement away from the equilibrium position and time satisfy the certain relation where x=Acos(ωt+φ). Suppose the mass of particle is m, then the kinetic energy is:

(3) $$E_{k}=\frac{1}{2}mv^{2}=\frac{1}{2}m\omega^{2}A^{2}\sin^{2}(\omega t+\varphi )=\frac{1}{2}kA^{2}\sin^{2}(\omega t+\varphi )$$

and the potential energy is:

(4) $$E_{p}=\frac{1}{2}kx^{2}=\frac{1}{2}kA^{2}\cos^{2}(\omega t+\varphi )$$

The total energy of the particle should require that $E=E_{k}+E_{p}=\frac{1}{2}kA^{2}$. So, the energy of a vibration particle should be proportional to the square of amplitude. The whole energy of a vibration signal should be proportional to the square of a kind of “amplitudes”. The energy features are those amplitudes. In fact, the square of such energy features are kinds of energy with a certain physical meaning.

As for feat7, this feature measures the mean value of frequency spectrum. According to Parseval’s theorem, we have the equation that $\frac{1}{N}\sum_{n=1}^{N}x_{i}^{2}=\sum_{k=1}^{K}{\left(s(k)\right)}^{2}$. So, feat7 presents a kind of “amplitude” too.

**Figure 5:**
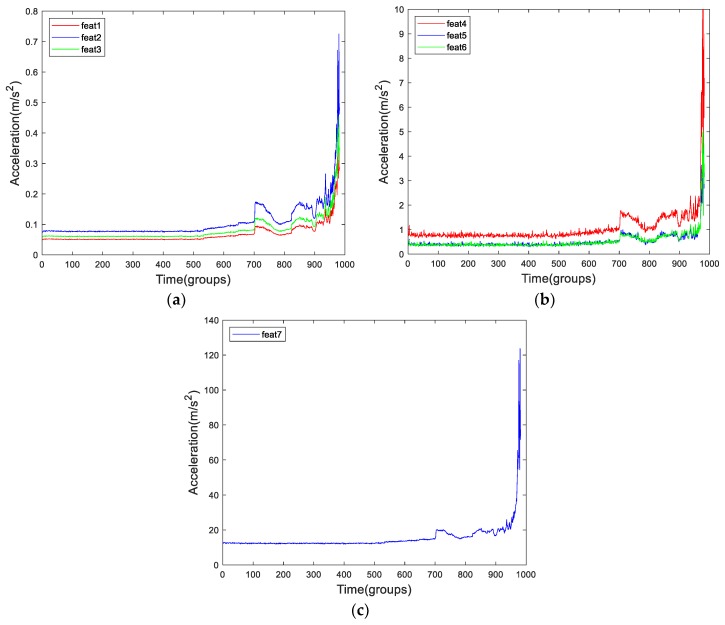
The curves of feat1 to feat7 of Bearing2-1IMS. (**a**) feat1 to feat3, (**b**) feat4 to feat6, (**c**) feat7.

Since each energy feature measures a certain “amplitude”, then there must be some connection between them. This connection leads them to have some sort of similarity. Figure 5 shows the feat1 to feat7 of Bearing2-1IMS. We can see there is a trend similarity between them. Owing to the calculation method of feat4, its values are approximately twice those of feat5 or feat6.

In Section 2, we introduced cointegration. If two time-series have a cointegration relationship then they have the same trend. First, we have to know the order of integration. Through the ADF test, we can determine that feat1 to feat3, and feat7 are I(2). Feat4 to feat6 are I(1). Feat4 to feat 6 all measure the extreme point of each group. They lack the ability of anti-jamming. Meanwhile, they lack of the ability to represent the signal of each group. Feat1 to feat3, and feat7, all measure the “average” amplitude of each signal. So, the curves of these features show a smoother appearance. Through the cointegration test, feat1 to feat3 and feat4 to feat6 have a cointegration relationship each other by setting the significance level as 0.1. The result is shown in Table 2. So, feat1 to feat3 have the same trend, meanwhile, feat4 to feat6 have the same trend when the significance level was set as 0.1. However, there is no cointegration relationship between the RMS and feat7. The reason is probably due to the shortcoming and limitation of FFT. Actually, the FFT has its disadvantages and is not suitable for nonstationary signals. So, the features extracted through FFT are unauthentic and biased.

**Figure 6:**
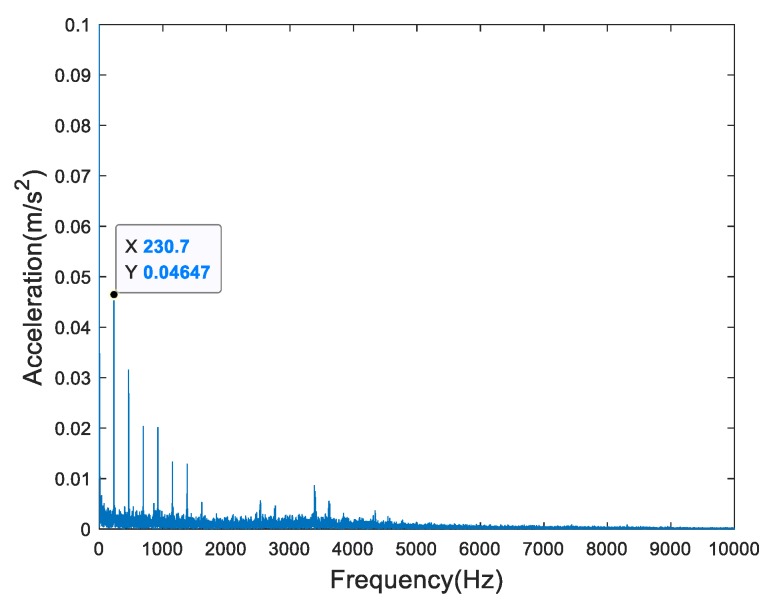
The frequency spectrum of the 700th group of Bearing2-1IMS demodulated by HT.

**Figure 7:**
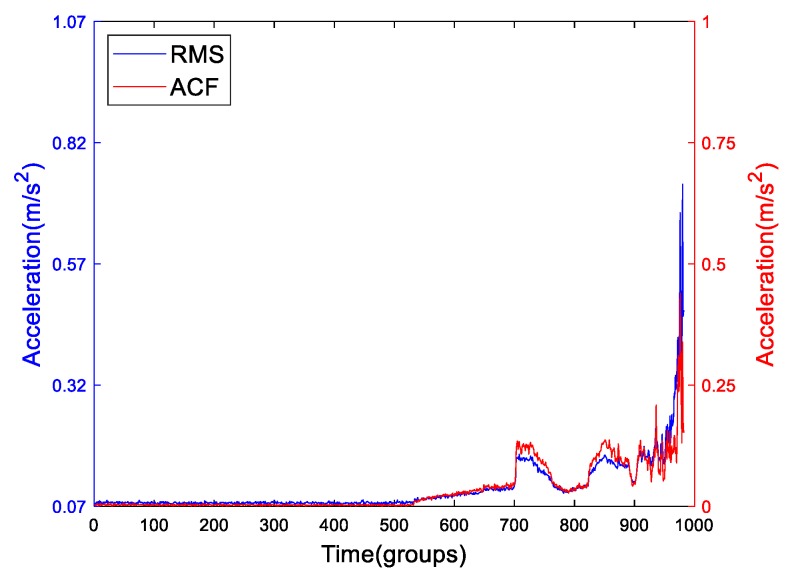
The ADF of Bearing2-1IMS compared with RMS.

In many references, many signal processing methods have been applied to process the run-to-failure data, e.g., EMD, LMD, VMD, EWT, etc. What these references have in common is that they use many signal processing algorithms to find the defect or characteristic frequency. In a few of references, the authors used the amplitude of characteristic frequency (ACF) as a degradation feature which is shown in Table 1 as feat8. In Figure 6, we plot the frequency spectrum of the 700th group of Bearing2-1IMS demodulated by Hilbert transformation (HT). As it shows, the outer race defect which values 230.7 Hz is obvious, and its amplitude is 0.04647 m/s^2^. Then we extract every characteristic amplitude of each group of the signals demodulated by HT to form the ACF as shown in Figure 7 compared with RMS. We can see that there is a similarity between both, but at the end of failure, the growth rate of ACF is not as fast as RMS. For ACF, it just measures the variation trend of the amplitude of fundamental defect frequency of the run-to-failure data. For RMS, it measures the average amplitude of each group. As Figure 6 shows, the spectrum exists septuple frequency at least which means that the fundamental frequency is not all the defect frequencies. Moreover, the bearing’s defect not only includes pitting or dent but also wear or abrasion. The wear may not generate defect frequency. Nevertheless, it is an important component of bearings’ degradation which cannot be ignored. So, the ACF could not reflect the holistic bearings’ degradation process correctly.

After the comparation and analysis, we can see that each energy feature represents an “amplitude”. They have advantages and disadvantages. For feat7 and feat8, they have their essential demerits. For feat4 to feat6, they can measure the extreme point of each group’ signal, however, each approach is lack of anti-jamming ability. For feat1 to feat3, since they have cointegration relationship each other, then they have the same trend. Due to the RMS is widely used, so we choose it to be the representive of energy features.

### 3.2. Complexity Features

Most of complexity features are downtrend with the deepening of bearings’ degradation. In this paper, we are going to compare and analyze the six common used complexity features. They are Shannon Entropy (ShEn), Approximate Entropy (ApEn), SampEn, Fuzzy Entropy (FuzzyEn), Permutation Entropy (PermEn), Lempel-Ziv Complexity (LZC), respectively. The ShEn is first proposed complexity which was developed in 1948 [22]. A problem with ShEn is that it is relatively insensitive to the changes in the tails of the distribution. In 1976, Lempel and Ziv proposed a specific algorithm of complexities called LZC [23]. ApEn is first developed by Pincus to handle the limitations that accurate entropy calculation requires vast amounts of data and great influence by system noise in 1991 [24]. SampEn is a modification of ApEn proposed by Richman and Moorman in 2000 [25]. It has two advantages over ApEn: data length independence and a relatively trouble-free implementation. Besides, there is no template vector comparison between itself in SampEn. In 2002, Bandt and Prompe introduced PermEn which based on comparisons of neighboring values of times series [26]. FuzzyEn is another modification of ApEn and it extended the “membership degree” with a fuzzy function [27].

The calculation procedures of the six complexities were illustrated of each reference in detail. They have connection each other. SampEn and FuzzyEn are two modifications of ApEn in different aspects. PermEn have somewhat similar in form to ApEn, e.g., phase-space reconstruction, but it calculates complexity in ShEn form. As to LZC, it is a complexity who has to coarse-graining. To compare the performance of the sixes, we need to minimize the influence of parameters, and the communal parameters should be set consistently. The setting of parameters is within the recommended values of their original references. Particularly, though PermEn has the phase-space reconstruction procedure, which has the same form as ApEn, FuzzyEn and SampEn. However, they are essentially different. For PermEn, vectors come from the phase-space reconstruction are not to be compared. Comparisons exist within the vectors. For the other three, comparisons are implemented between vectors to calculate the distance. So, the embedding dimension m of PermEn is different from the others. Large m will extremely increase the calculation time, and we set m=6. All the selected parameter values are presented in Table 3, where *std* is the standard deviation of the signal. In particular, when calculating ShEn, it needs to acquire the probability distribution function (PDF) of the signal. The easy way to estimate the PDF is to use the histogram where the amplitude range of the signal is linearly divided into *k* bins so that the ratio k/N is constant. The ratio k/N characterizes the average filling of the histogram.

**Figure 8:**
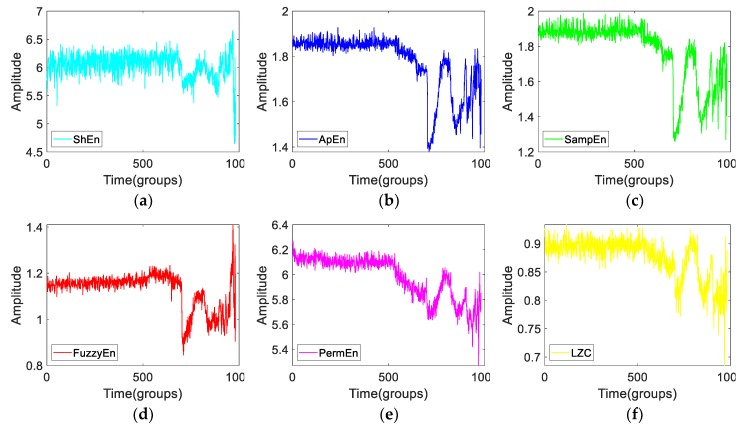
The six complexity features of Bearing2-1IMS. (**a**) ShEn, (**b**) ApEn, (**c**) SampEn, (**d**) FuzzyEn, (**e**) PermEn, (**f**) LZC.

Figure 8, shows the six complexity features of Bearing2-1IMS. From Figure 8, we can see that there is a trend similarity between them. However, complexities are different in performance. Next, we are going to find out which of them has the best performance when measuring the vibration signals by using simulation signals.

• Performance in sinusoidal signals with additive white noise

**Figure 9:**
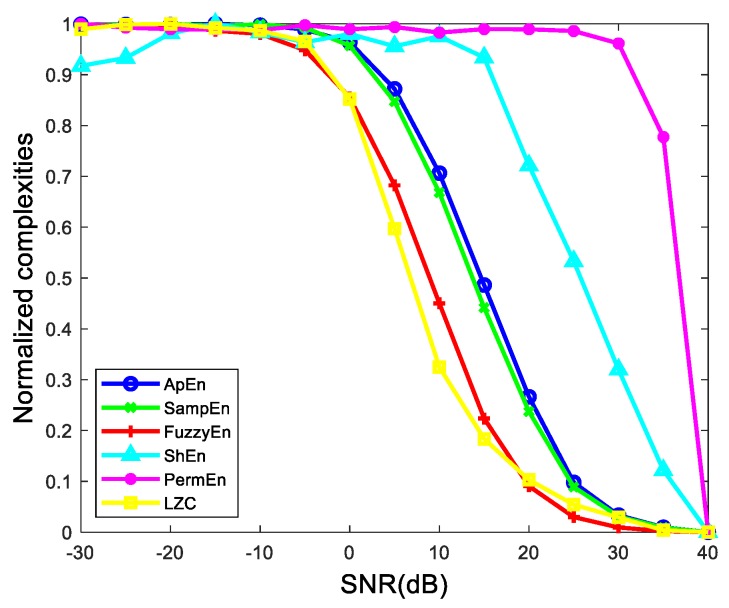
The curve of six complexities versus SNRs.

To test the performance of complexities, the first thought is to test in periodical signals with different intensity noise. And we give a group of simulation signals, S(t)=X(t)+e(t), where X(t)=sin(2π×10t) and e(t) is the additive white noise. The sampling frequency is 10,000 Hz with 1 s duration. Figure 9 shows the complexities with different SNRs. For convenience, all the complexities have been normalized.

We can see that all the complexities tend to ascend with the increasing of the additive white noise. Among the sixes, ShEn and PermEn are the worst, since they do not have a good monotonous tendency. The rests present good monotonicity.

• Performance in logistic map

**Figure 10:**
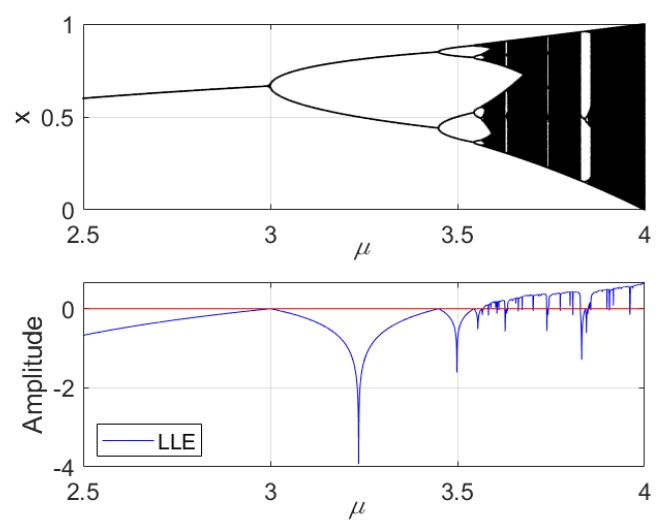
The logistic map and its LLE.

In above, we have discussed the performance of the six complexities in a group of simulation signals. In this part, we are going to use more general simulation signals. Thus, the logistic map was taken as an example, since it can generate periodical and chaotic signals. As many references shown, rolling bearings are highly nonlinear components and many studies have reported nonlinear phenomena such as bifurcations, quasi-periodicity, and chaos in rolling element bearings [28,29,30]. Logistic map, as is well known, is a nonlinear dynamic system, where a simple mathematical model is used to describe the change of insect quantity with time increasing. Logistic map can be written as $x_{n+1}=\mu x_{n}(1-x_{n})$. Figure 10 shows the bifurcate evolutional process of the logistic map where 2.5<μ<4, meanwhile, Figure 10 shows the largest Lyapunov exponent (LLE) of the logistic map. Where LLE < 0 means the system is periodical. LLE = 0 means bifurcation occurs; and LLE > 0 means chaotic behaviors occur. As is known, the period 2 bifurcation occurs at μ=3; the 4 bifurcation occurs at μ=3.449. After μ=3.83, there is a short period 3.

**Figure 11:**
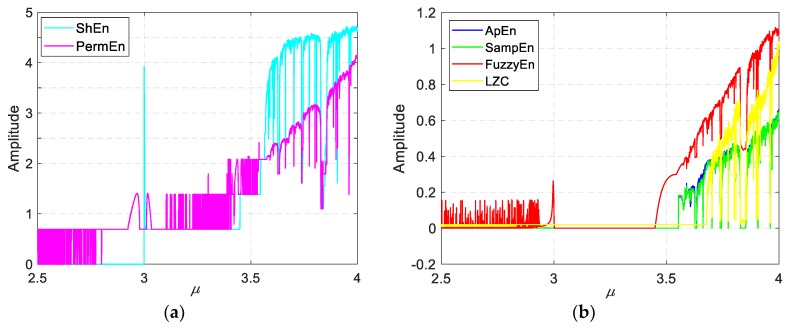
The six complexities of the logistic map. (**a**) ShEn and PermEn, (**b**) ApEn, SampEn, FuzzyEn and LZC.

Similarly, we have calculated the six complexities at the case of logistic map, and the results are shown in Figure 11. As we can see, PermEn has quite a few fluctuations before μ=3.5. ShEn has some steps before μ=3.449. FuzzyEn has some fluctuations before μ=3 and something wrong at μ=3.5. The reason of that must lie in the membership function of the FuzzyEn, in other words, since the membership function of FuzzyEn, the measurement of the boundary between period and chaos is not very clear. LZC has some wrong value around μ=3.6, where there exist chaotic behaviors, but its value is close to zero. All the sixes exhibit the short period 3 after μ=3.83. In this part, ApEn and SampEn have the better performance.

• Performance in rate of convergence

**Figure 12:**
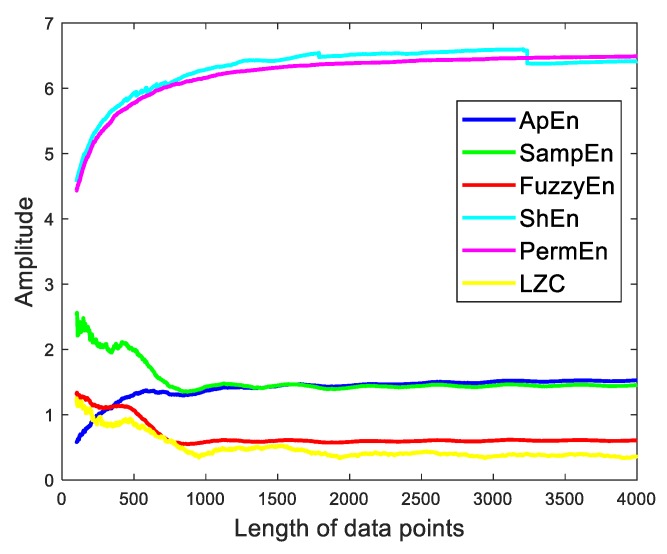
The curves of six complexities versus length of data points.

The complexities are also affected by the length of data. It is inaccurate to use the data which the complexities are not convergent. So, to make clear the performance in rate of convergence are another comparison of complexities. Taking a sinewave signal S(t) with −10 dB addictive white noise as a case, the complexities values are calculated from the length 100 to 4000 as illustrated in Figure 12. The PermEn and ShEn have similar increasing convergence trend, since the core operation of PermEn is ShEn. And the complexities values are convergent after 2000 data points. SampEn, FuzzyEn, LZC have similar convergence trend. The complexities values have a fluctuant decreasing trend. ApEn is different from the above, and it has a fluctuant increasing trend. From the comparison of convergence rate, we can confirm that ShEn and PermEn are no doubt the worst.

As mentioned above, we have used three methods to compare the six complexities. Among them, ApEn and SampEn have the better performance. As an improvement of ApEn, SampEn is more authentic and accurate. And it can be a representive feature of complexities.

## 4. The Novel Health Indicator Based on Cointegration

In Section 3, we have discussed the energy features and complexity features. And we have concluded that the RMS and SampEn are two representatives of the two subdivisions. Back to the Figure 1 in the Introduction, there may exists cointegration relationship between the RMS and SampEn. In this section, we are going to explore whether there is a cointegration relationship between them and will propose the novel health indicator based on cointegration theory.

**Figure 13:**
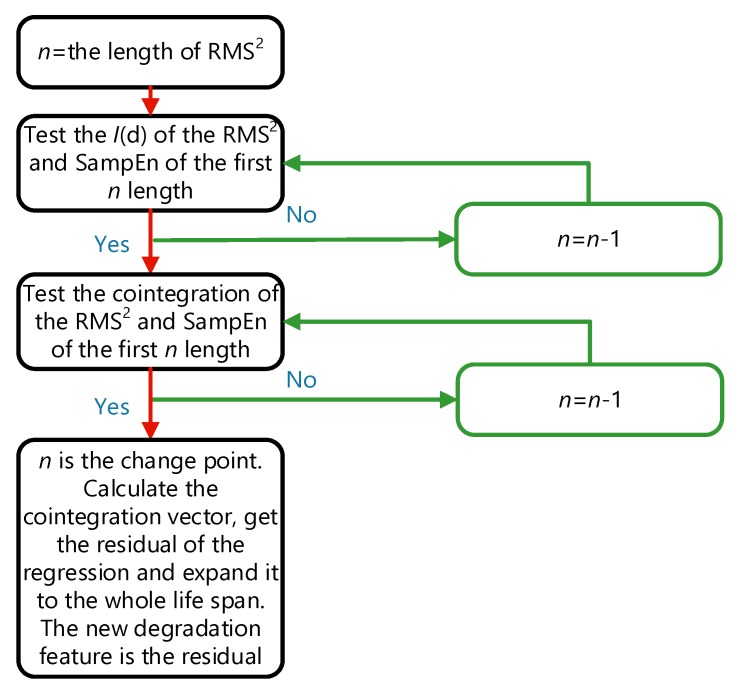
The flow chart of extraction of the new degradation feature.

**Figure 14:**
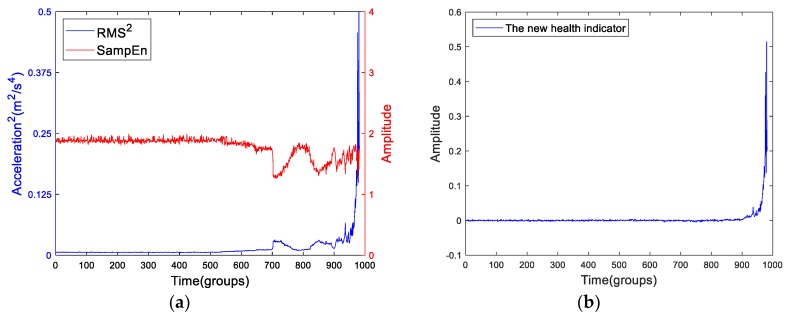
(**a**) The RMS^2^ and SampEn curves of Bearing2-1IMS and (**b**) the new health indicator.

First, we have to confirm that the squares of the energy features represent the energy. And we define the term RMS^2^ as the square of the RMS. By using the ADF test, we found that the RMS^2^ is I(2), but SampEn is I(1). However, the basic requirement of cointegration where the order of integration of two time-series should be the same has not been met. Next, we can gradually reduce the length of RMS^2^ and SampEn by removing the last point and check the order of integration of the two truncated features. The iteration is stopped once the two truncated features satisfy that they have a cointegration relationship at a given significance level (e.g., 0.1). The stop point *n* is defined as the change point. In this example, the change point is 914. The proposed health indicator is the extend residual of the cointegration regression. Figure 13 shows the flow chart of extraction of the new health indicator. Figure 14 shows the RMS^2^, the SampEn and the new health indicator of Bearing2-1IMS.

From the Figure 14b, the new health indicator shows a straight appearance before n=914, and then the curve rises rapidly. The curve clearly exhibits a character of “two-stage” property. Before n=914, the curve is stationary, and the curve presents like a straight line. When n>914, the curve goes up like in exponential form. As shown in Figure 14a, the RMS^2^ raises up when the bearing is close to failure. At this period, the SampEn doesn’t change a lot. The reason of this phenomenon will be discussed in detail.

**Figure 15:**
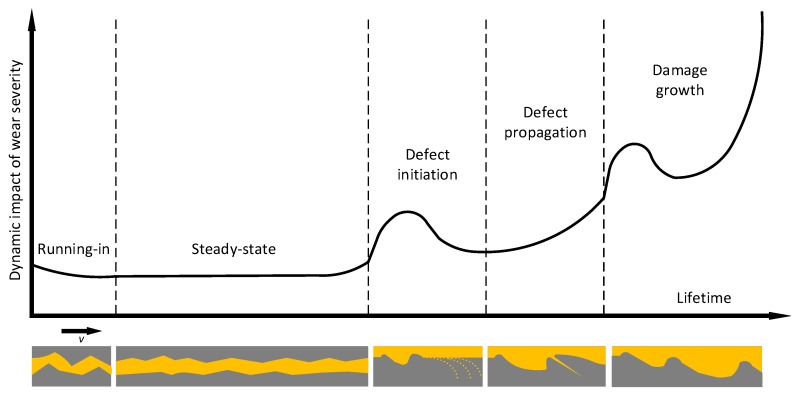
The descriptive model proposed by El-Thalji and Jantunen.

The degradation process of rolling bearings is complex. In fact, the degradation of rolling bearings is an evolution of wear. El-Thalji and Jantunen have built a descriptive model of wear evolution [31,32]. Figure 15 shows the descriptive model which has five-stage scenario, namely running-in, steady-state, defect initiation, defect propagation, and defect growth. It can help to have a quick review of the evolution of the degradation of wear.

There are some degrees of surface roughness and waviness of newly manufactured bearings. Therefore, the surface asperities will interact with each other in the running-in stage. This stage is usually short and inconspicuous. When the surface asperities are smoothened by cycles and the lubrication film become uniform, the bearing is running into the steady-state stage, i.e., the healthy stage. Over time, high stress, contamination particles, vibration and lubrication disturbances will result in asperities and dents. At this time, the defect zone includes the leading edge asperity, the dent and trailing edge asperity along the rolling direction. When the rolling element passes the defect zone, the double-impulse phenomenon will occur. At the same time, the asperities will be reduced due to the over-rolling and abrasive wear. This is the so-called “healing” or smoothing phenomenon. The over-rolling usually flattens the top of the asperity. Thus, it forms a plastic deformation contact around the dent. Although the asperities are smoothed, the over-rolling cycles will impose enough applied stress and large enough variation to the material to initiate and accelerate the crack opening. Now, the bearing is in the defect initiation stage. When the crack is opened, the stress centration points will shift to the tip of the crack. The defect will propagate. Later, the crack continues expansion and eventually becomes parallel to the surface. Then, a secondary crack connecting the former crack to the surface results in pits and debris. When the defect propagation stage ends, a complete defect is formed and the bearing is running to the last stage, the damage growth stage. In this stage, the spalling phenomenon occurs. The spalling is the advanced stage of flaking. In this stage, the wear condition becomes fierce and massive abrasive particles come out. The curve of wear becomes rapid increased. The crack actions, the over-rolling and abrasion will make new defects and lead to more debris particles, making the process fiercer. The wear process is like a chain reaction which causes the bearing fail quickly. In this model, two “healing” phenomena can be seen.

**Figure 16:**
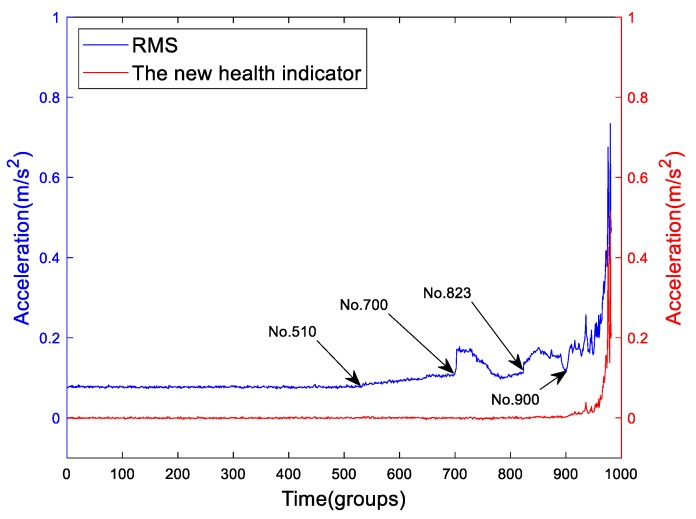
The RMS and the proposed health indicator of Bearing2-1IMS.

The wear evolution is a nonlinear process. The RMS can represent the average energy of bearings which can also reflect the wear evolution indirectly. For convenience, we put the RMS and the new health indicator of Bearing2-1IMS together as shown in Figure 16. The running stage of Bearing2-1IMS is inconspicuous or almost non-existent. Before 510th group, the RMS stays steady. From 510th to 700th groups, the RMS is linear, which means the asperities on the interaction surface are becoming more and increasing in size. The 700th group is a transition point. At this point, the RMS experiences a mutation which probably indicates that the crack is opened. From the 700th to 823th groups, the first “healing” process occurrs. In this stage, the defect is propagating. From 823th to 900th groups, the RMS experiences the second visible “healing” process. The 900th point is the minimum point of the second “healing” process. From the 900th to the end, the RMS grows quickly with fluctuation. It can be seen that the RMS of Bearing2-1IMS is good reflection of the descriptive model proposed by El-Thalji and Jantunen.

The trend of RMS is fluctuant since the wear evolution is a nonlinear process. However, the trend of the proposed health indicator is not as fluctuant as the RMS. It shows a “two-stage” character. Before the 914th group, it exhibits as a zero slope line. After the 914th, it raises quickly. Actually, the change point lies in the last stage of the descriptive model proposed by El-Thalji and Jantunen. The change point is close to the 900th point which is the minimum point of the second “healing” process of the RMS. So, the new health indicator can solve the “healing” phenomenon problem that is expressed by RMS which is caused by the wear evolution. It is to be noted that the fluctuation caused by “healing” cannot be eliminated through denoising methods.

When a rolling bearing is running, if there is a local asperity of the rolling bearing, the energy will be concentrated, thus making the energy increase. At the same time, the periodical impact signals are observed, thus making the RMS increase and the complexity decrease. This increasing and decreasing are synchronous even if there is a “healing” process. This synchronization shows cointegration of the curves of RMS^2^ and SampEn. When there is a smoothing phenomenon, the defect is smoothened due to rolling effect and abrasion, here the amplitudes of the periodical impact are reduced, therefore, the RMS decreases. As the difference between impact amplitudes and base noise amplitudes are reduced, the complexity increases. However, the last stage of wear evolution is characterized by multi/distributed and large defects, so the impact amplitudes become higher and stronger. Each defect produces periodic impacts, but the holistic measured signals look not so periodical, which means the difference between the impact amplitudes and overall signals are not that high, so the complexity decreases but not to a large extent. If the defect is a single defect until the failure, then the RMS^2^ and complexity are synchronous all the time. However, the defects of bearings close to failure are always multiple. So, we can consider that the defects of a bearing become multiple and distributed after the change point. Here, the vibration signal is a result of multiple defects combination. When the defects are multiple and distributed, it means the bearing is close to failure. So, the change point can be deemed as the first predicting point to prognosis. Besides, monotonicity is an index which can measure the ability of prognostics [33]. Apparently, the non-subtractive property of the new health indicator is higher than the RMS^2^.

Moreover, by carefully studying Figure 16, if we separately choose the 703th and 775th groups which present the local peak at the initial point and local trough of the first “healing” phenomenon of the Bearing2-1IMS for diagnosis, it is easy to give a wrong result that the fault degree of the 703th is more serious than the 775th. Due to the “healing” phenomenon, the results of diagnosis and run-to-failure monitoring have some differences. So, the result of run-to-failure test is more realistic than the preset test, since the preset test cannot simulate the “healing” process.

**Figure 17:**
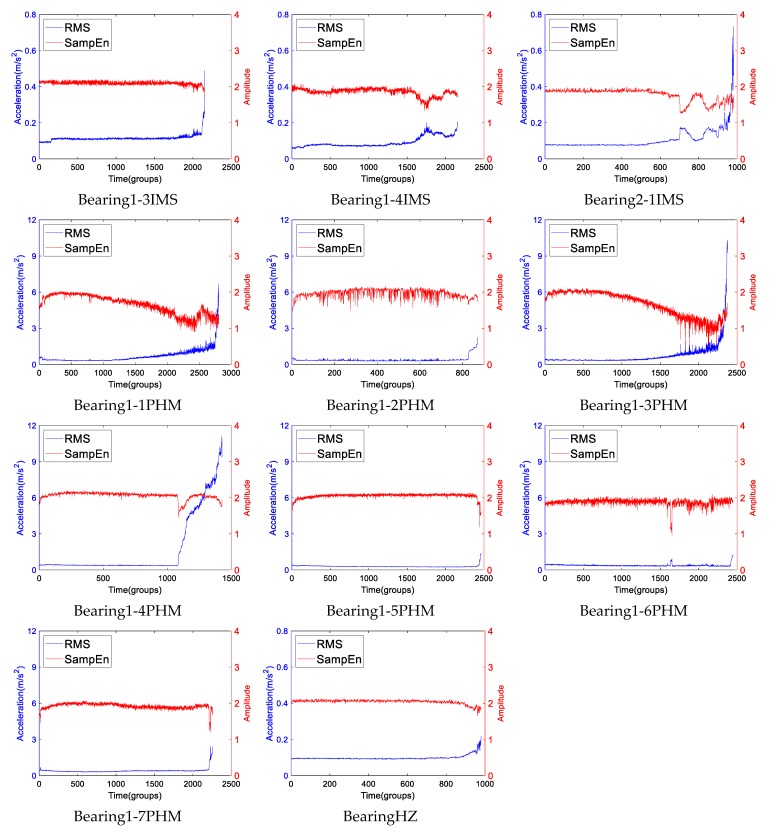
The RMS and SampEn curves of different run-to-failure rolling bearings’ data.

This “two-stage” character of the proposed indicator is a general property of bearings. To give a better impression, Figure 17 shows the RMS and SampEn of a lot of different run-to-failure rolling bearings’ data which are elaborated in the Appendix A. As we can see, there are eleven bearing’s run-to failure data which come from three datasets. The RMS of most data shows no distinct “healing” phenomena, but distinct fluctuations during the middle or late period of lifetime, i.e., Bearing1-1PHM, Bearing1-2PHM, Bearing1-3PHM, Bearing1-6PHM. Some RMSs appear very stable until the quick increase close to the end, i.e., Bearing1-3IMS, Bearing1-5PHM, Bearing1-7PHM and Bearing HZ. The Bearing1-4IMS and Bearing2-1IMS exhibit distinct “healing” phenomena. In each example, the RMS and SampEn look inversely similar.

**Figure 18:**
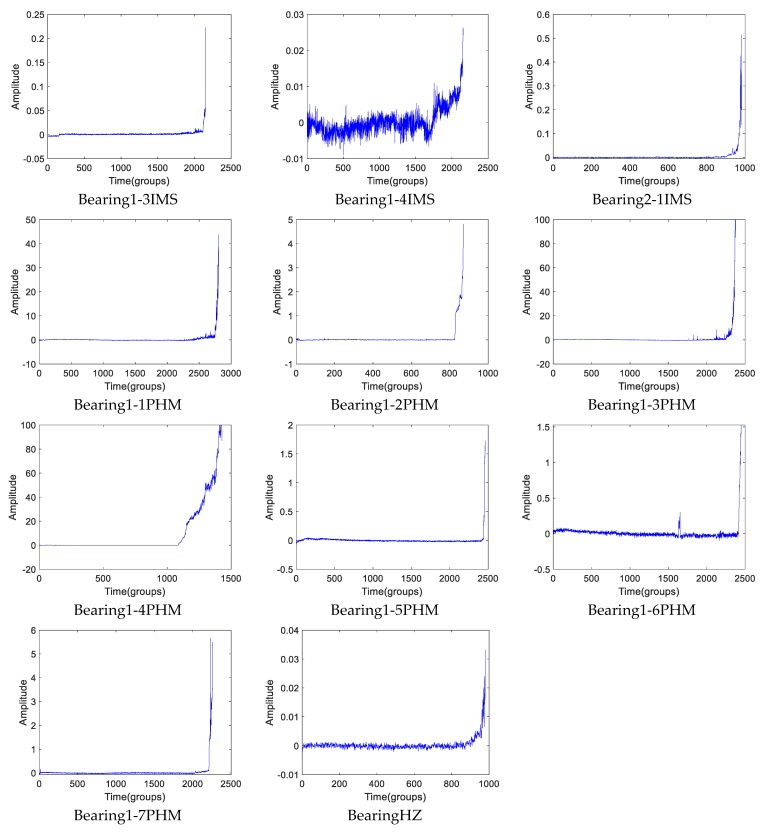
The novel health indicator of different run-to-failure rolling bearings’ data.

By cointegration, the new health indicators of the eleven data are extracted, which are shown in Figure 18. As we can see, each proposed health indicator shows distinct “two-stage” character, the zero-line stage and quickly raise stage. Take a look at the results of Bearing1-3IMS of Figure 17 and Figure 18, we can see that there is an inshot at the 156th point of the RMS and a slight uplift of its new health indicator. By checking the raw data of IMS Dataset I, we can find that the experiment is not continuously operating, the test is discontinuous, and the 156th is an interrupting point. The condition of the test may have changed at such point. It gives us an enlightenment that the run-to-failure test should be continuously completed. Shutdown or discontinuous operation may change the authenticity of data. There is more noise in the results of Bearing1-4IMS and BearingHZ. It is appropriate to use denoising methods before or after the fuse process.

The zero-line stage of the “two-stage” character can be simulated by a straight line. The quick raise stage can be simulated by an exponential function. For the second stage, it is a period that the number and scale of defects are increasing. Cracks are occurred between the adjacent defects and make them merged into a big defect, thus making more spalling. The crack propagation can be estimated by the Paris law which indicates that the rate of crack growth shows exponential form. At this stage, the health indicator is mainly affected by the RMS. The RMS presents an exponential form. So, the exponent can be used to simulate the quickly raise stage.

There are many degradation models for modelling the degradation process of bearings. The exponential degradation model is one of the most popular methods for modelling the bearing’s degradation. As for the “two-stage” character of the proposed indicator, it is easy to associate with the exponential degradation model. It was first established by Gebraeel et al. in [34]. The model is depicted as follows:

(5) $$\begin{array}{ll} S(t_{i}) & =\phi +\theta \exp(\beta t_{i}+\varepsilon (t_{i})-\frac{\sigma^{2}}{2})\\ & =\phi +\theta \exp(\beta t_{i})\exp\left(\varepsilon (t_{i})-\frac{\sigma^{2}}{2}\right) \end{array}$$

For i=1,2,⋯, where ϕ is a constant, θ is a lognormal random variable, namely lnθ is yielding to $N(\mu_{0},\sigma_{0}^{2})$. β is a normal random variable which is yielding to $N(\mu_{1},\sigma_{1}^{2})$. $\varepsilon (t_{i})$ is a random error term that follows a normal distribution with mean zero and variance $\sigma^{2}$. And ϕ, β and $\varepsilon (t_{i})$ are assumed mutually independent and $\varepsilon (t_{1})$, $\varepsilon (t_{2})$, …,$\varepsilon (t_{i})$ are independent identically distributed random variables. The existent term $\sigma^{2}/2$ is to make that $E[\exp(\varepsilon (t_{i})-(\sigma^{2}/2))]=1$ and thus $E[S(t_{i})|\theta ,\beta ]=\phi +\theta \exp(\beta t_{i})$.

The exponential degradation model can be used to estimate the RUL of the bearings. In [34], RMS is chosen as the degradation feature for the exponential degradation model. However, as Figure 17 shows, there are many differences of each RMS. It is easy to come up with a query whether the exponential degradation model is more suitable for RMS or the proposed indicator. Next, we are going to have a discussion.

**Figure 19:**
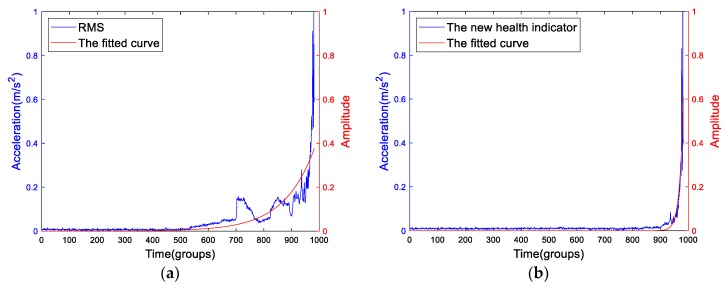
The smoothed and normalized RMS and the proposed indicator. The (**a**) normalized RMS and (**b**) the normalized new health indicator and their fitted curves.

The features should be normalized. By using the exponential fitting method, the fitted curves of Bearing2-1IMS are shown in Figure 19. It is easy to see that the proposed health indicator is more suitable for the exponential model. The SSE, R-square and RMSE of the RMS’ fitting are 2.228, 0.706 and 0.04768. The result of the proposed health indicators are 0.7078, 0.7655 and 0.02687. So, the result of the proposed health indicator is better, which means that the exponential degradation model is more suitable for the proposed indicator than RMS.

## 5. Discussion

In this section, we are going to discuss two issues about the proposed method. The first is the significance of the proposed indicator. Accurately, cointegration is a fusion method. The second is the similarities and differences of between the cointegration fusion method and other fusion methods.

### 5.1. The Significance of the Cointegration Fusion Method

In general, it is not advisable to fuse features of different properties. However, by using the cointegration test, it can be observed that the RMS^2^ and SampEn have a sort of cointegration relationship. So, actually, the two features can be fused. Meanwhile, the method solves a problem in the difference between the energy features and complexity features. The cointegration is a bridge of the energy features and complexity features. We have found the change point is the transition of local defects and distributed defects. By using cointegration, the problem of fluctuations due to the “healing” process was resolved.

### 5.2. The Comparison between Cointegration Fusion Method and Other Fusion Methods

**Figure 20:**
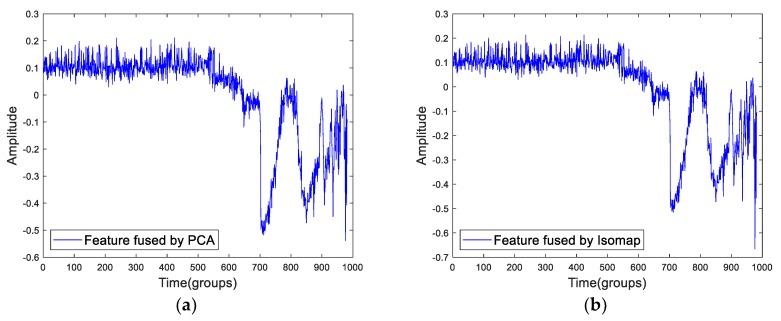
The fused feature of RMS^2^ and SampEn based on (**a**) principal component analysis (PCA) and (**b**) Isometric Feature Mapping (Isomap).

In essence, in this paper, the cointegration is regarded as a fusion algorithm to fuse the RMS^2^ and SampEn. So, we must compare the cointegration method and other fusion methods to find what in common and what in difference. The principal component analysis (PCA) and Isometric Feature Mapping (Isomap) are the commonly used fusion methods or dimensionality reduction algorithms. Particularly, Isomap is a typical method of manifold learning. Figure 20 shows the fusion result of Bearing2-1IMS’ RMS^2^ and SampEn using by PCA and cointegration.

As we can see from the Figure 20, the results of PCA and Isomap look similar. Meanwhile, the results are similar to the SampEn. In fact, the PCA and Isomap are finding data that the features have in common. However, the cointegration is a fuse method that finds the difference between features. Certainly, the information of the proposed health indicator is limited. The first predicting time of the proposed indicator is later than RMS. When predicting the RUL, it is better to combine more information.

## 6. Conclusions

In this paper, we have discussed the energy and complexity features of rolling bearings. We found that the RMS and SampEn are two representatives of the two subdivisions. By using the cointegration theory, we have proposed a new health indicator. The indicator has “two-stage” character which can divide the lifetime of bearing into two stages, one is the zero-line stage, the other is the exponential quick raise stage. We have explained the cause of the two stages. Additionally, we have used eleven run-to-failure data of rolling bearings to verify the general property of the proposed method. It is more suitable for the proposed indicator to use the exponential degradation model.

## Figures and Tables

**Table 1:** Some energy features.

$feat1=X_{r}={\left[\frac{1}{N}\sum_{n=1}^{N}\sqrt{\left|x_{i}\right|}\right]}^{2}$	$feat2=X_{\mathrm{rms}}=\sqrt{\frac{1}{N}\sum_{n=1}^{N}x_{i}^{2}}$	$feat3=\mu_{\left|x\right|}=\frac{1}{N}\sum_{n=1}^{N}\left|x_{i}\right|$
$feat4=X_{p-p}=\max(x_{i})-\min(x_{i})$	$feat5=\max\{\left|x_{i}\right|\}$	$feat6=-\min\{x_{i}\}$
$feat7=\frac{1}{K}\sum_{k=1}^{K}s(k)$	feat8=ACF	
Where $x_{i}$ is a signal series for i=1,2,⋯,N, N is the number of the data points. Where s(k) is a spectrum for k=1,2,⋯,K, K is the number of spectrum lines, s(k)≥0.		

**Table 2:** The Engle-Grangertest results of energy features.

Dependent Variable	Tau-Statistic	Prob.
feat1	−2.623079	0.0701
feat3	−2.535995	0.0852
feat7	−2.278023	0.1448
dependent variable = feat2		
feat5	−7.597659	0.0000
feat6	−7.515313	0.0000
(dependent variable = feat4)		

**Table 3:** The summary of the selected parameter values.

Complexities	Parameters	Value
ShEn	Average filling of the histogram, k/N	0.02
ApEn	Embedding dimension, m	2
	Tolerance, r	0.2⋅std
	Delay time, τ	1
SampEn	Embedding dimension, m	2
	Tolerance, r	0.2⋅std
	Delay time, τ	1
FuzzyEn	Embedding dimension, m	2
	Tolerance, r	0.2⋅std
	Parameter, w	2
	Delay time, τ	1
PermEn	Embedding dimension, m	6
	Delay time, τ	1
LZC	Coarse-graining accordance	median value

**Figure A1:**
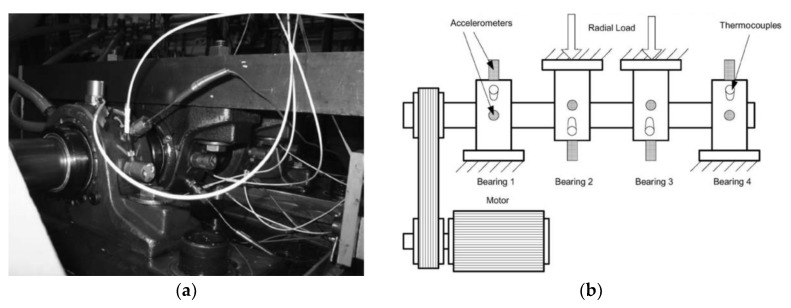
Bearing test rig of run-to-failure dataset of IMS center. (**a**) actual diagram (**b**) schematic diagram.

**Figure A2:**
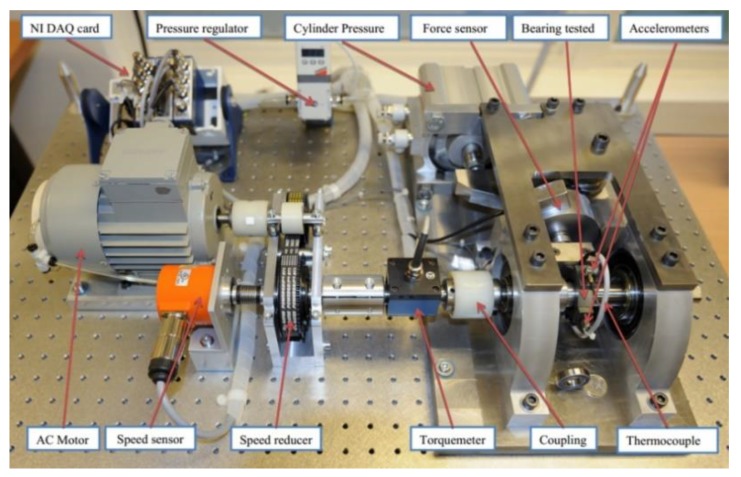
Overview of PRONOSTIA.

**Figure A3:**
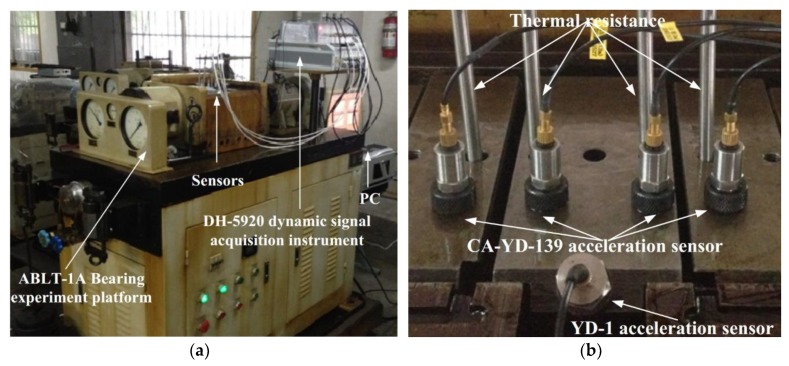
(**a**) The experimental rig and (**b**) the sensors layout of Hangzhou Bearing Test and Research Center.

## Appendix A. The Bearings’ Run-To-Failure Datasets Used in the Paper

### Appendix A.1. Dataset I: The Run-To-Failure Dataset of IMS Center

The Dataset I is from IMS center [5]. The bearing test rig is shown in Figure A1 which hosted four test bearings on a shaft driven by an AC motor. The rotation speed kept constant at 2000 rpm. A constant radial load of 6000 pounds was added to the shaft and bearings by a spring mechanism. All the bearings were force oil-lubricated. Four Rexnord ZA-2115 double row bearings were installed on the shaft. The bearings had 16 rolling elements in each row. The pitch diameter is 2.815 inch, and the roller diameter of 0.331 inch. The tapered contact angle is 15.17°. A high sensitivity acceleration sensor was installed on each bearing’s housing. The data sampling rate was 20 kHz and the data length were 20,480 points (For saving time, we use the former 4000 points to calculate features). The sampling interval was 10 min.

In the document of this dataset the failure mode of the bearing 1 of Set No. 2 (short for Bearing2-1IMS) is outer race fault. This data is used through the beginning to the end of the paper. Besides, the Bearing1-3IMS which presented inner race fault and the Bearing1-4IMS which presented roller element fault are used in the paper.

### Appendix A.2. Dataset II: The Run-To-Faliure Dataset of IEEE PHM 2012 Prognostics Challenge

The Dataset II comes from the IEEE PHM 2012 Prognostics Challenge, which was provided by the FEMTO-ST Institute [35]. The challenge was focused on the estimation of the remaining useful life of bearings. Figure A2 shows the experimentation platform which is called PRONOSTIA. In this dataset, 3 different loads were considered:

- First conditions: 1800 rpm and 4000N;
- Second conditions: 1650 rpm and 4200N;
- Third conditions: 1500 rpm and 5000N.

The participants of the Challenge are provided with six complete run-to-failure datasets to build the prognostics models. There are 11 truncated datasets which were asked to estimate the remaining life and verify the prognostics models. To avoid propagation of damages to the whole test bed (and for security reasons), tests were stopped when the amplitude of the vibration signal overpassed 20 g. In the Dataset I, we knew some failure types of some datasets. But in the Dataset II, no assumption about the types of failure were given. The bearings had 13 rolling elements and the diameter was 3.5 mm. The pitch diameter of the experiment bearing was 25.6 mm and the tapered contact angle was 0°. The sampling frequency was 25.6 kHz and the data length of each group was 2560 points (i.e., 1/10 s). The sampling interval was 10 s.

In this paper, the seven bearings in first condition are used to verify the proposed method which are short for Bearing1-*x*PHM.

### Appendix A.3. Dataset III: The Run-To-Failure Dataset of Hangzhou Bearing Test and Research Center

The above two datasets are all public data. We also cooperated with Hangzhou Bearing Test and Research Center, China. The test was performed on four bearings with a same shaft. In order to achieve a smooth loading, the load was started only 5 kg and added 5 kg every 15 min. The maximum load was set to be 50 kg which meant that each bearing was under about 6.3 kN radial load. All the bearings were force oil-lubricated. If a bearing run to failed, a new one was then installed immediately to reduce the test time. A good monitoring and signal acquisition system was working during the whole process of the experiment. The experimental rig and the sensors layout are shown in Figure A3. There were four thermocouples and a YD-1 acceleration sensor. The thermocouples were used to monitor the temperature of each bearing. The YD-1 acceleration sensor was used to monitor the state of bearings. There are four CA-YD-139 acceleration sensors which were installed on the cover plate to acquire bearings’ vibration data. The data were transferred to the PC client through the DH-5920 dynamic signal acquisition instrument.

The type of bearings used in the experiment was 6204. The sampling frequency was 25.6 kHz. The sampling interval was 10 min. During the experiment, a threshold of failure was set through the monitoring system. There are two criteria to stop the test by experience. One is that the current RMS value is more than 2.5 times of the initial RMS value. The other is that the temperature is over 70 °C.

We have tested eight bearings and completely collected five bearings’ data. The data of No. 5 bearing is used to verify the proposed method in this paper. This data is also used in [36,37].
